# Global burden of hypertension in people living with HIV

**DOI:** 10.1186/s12916-021-01981-y

**Published:** 2021-05-14

**Authors:** Jean Joel Bigna, Jean Jacques Noubiap

**Affiliations:** 1 Department of Epidemiology and Public Health, Centre Pasteur of Cameroon, Yaoundé, Cameroon; 2 Centre for Heart Rhythm Disorders, University of Adelaide and Royal Adelaide Hospital, Adelaide, Australia

**Keywords:** Hypertension, Prevalence, HIV, AIDS

## Background

Due to increasing widespread use of highly active antiretroviral therapy (HAART) since 2000s, there has been a shift in the morbidity and mortality pattern of people living with HIV (PLHIV) from AIDS-related opportunistic infections towards age-related chronic diseases, including hypertension, diabetes, dyslipidemia, and cardiovascular disease (CVD) [1–4]. About 24% of PLHIV have hypertension, giving a total of nearly 8.9 million PLHIV who are affected globally [5]. Because these estimates vary substantially across regions and country level of income [5], disaggregation of data on the association between HIV infection and the risk of hypertension by region and country socioeconomic level is pivotal to better understand the interaction between both diseases and tailor context-specific interventions to curb their overlapping burden.

## Regional variability in the burden of hypertension in HIV

In *BMC Medicine*, Davis and colleagues present the first meta-analysis of the association between exposure to HIV infection and prevalent hypertension using data from 59 age-matched cross-sectional studies including more than 11 million participants [6]. Compared with HIV-uninfected individuals, the prevalence of hypertension was significantly higher in PLHIV in North America and lower in sub-Saharan Africa and Asia, whereas there was no significant difference in populations in South America and Europe. Although the authors did not investigate variability by country level of income, looking at the findings of this meta-analysis, one could hypothesize that there would be a significant increased risk of hypertension among PLHIV compared to HIV-negative in high-income countries (HICs) unlike low-income countries (LICs) where the risk would be reduced. Indeed, a previous meta-analysis revealed a higher prevalence of hypertension in North America and Western and Central Europe compared with sub-Sahara Africa, Asia, and the Pacific, although the number of PLHIV with hypertension was highest in sub-Saharan Africa as the burden of HIV in this region is the greatest [5].

## Why is there regional difference?

The association between HIV infection and hypertension is mainly determined by some biological factors. HIV and HAART (especially protease inhibitors and certain nucleoside and non-nucleoside reverse transcriptase inhibitors) promote microbial translocation, endothelial cell dysfunction, chronic inflammation, HIV-associated nephropathy, renin-angiotensin-aldosterone system activation, and arterial stiffness which altogether increase blood pressure in PLHIV [1, 2]. With the increased life expectancy due to HAART, longer exposure to HIV and HAART amplifies the risk of hypertension [1, 2]. Despite the increased HAART uptake by PLHIV in LICs such as those in sub-Saharan Africa, the mortality due to AIDS-related complications is still high compared to HICs. As a result, PLHIV in LICs have shorter life expectancy compared to their HIV-uninfected counterparts and, consequentially, have lower rates of age-related chronic conditions such as hypertension.

## What are the implications?

Interventions should be more aggressive towards modifiable risk factors (unhealthy diets—excessive salt consumption, a diet high in saturated fat and trans fats, low intake of fruits and vegetables; physical inactivity; consumption of tobacco and alcohol; and being overweight or obese) in PLHIV globally [7]. In LICs, especially those in Africa and Asia with the highest burden of HIV infection and the poorest indicators for HIV programs, the HIV care cascade (early HIV diagnosis, early HAART initiation and linkage to care, high level of linkage to care, and adherence to HAART) should continue to be improved, building on the encouraging achievements of past two decades [8]. Furthermore, there should be a more effective integration of services for HIV and non-communicable diseases including hypertension, diabetes, and dyslipidemia. Interventions for primordial cardiovascular prevention should be central in healthcare provision for PLHIV in these resource-limited settings, as there are more likely to be cost-effective. It is also crucial to ensure that all PLHIV have adequate access to screening and management of cardiovascular risk factors such as hypertension, considering the low level of awareness, treatment, and control of these conditions in the general population [9]. In the context of limited specialized healthcare personnel, task shifting represents a viable solution.

## Conclusion

The findings of the study by Davis and colleagues call for effective, intensified, and robust strategies for primordial prevention of cardiovascular risk factors among the global population of PLHIV. Adequate access to screening and management of cardiovascular risk factors such as hypertension should be implemented, particularly in Africa and Asia where most PLHIV live. Future research is needed to determine cost-effective interventions to reduce the burden of hypertension in PLHIV.

## Data Availability

Not applicable.
